# A new technology for a novel clinical approach in a dog with a complex vascular anomaly: the “extended reality”

**DOI:** 10.1007/s11259-025-10668-1

**Published:** 2025-02-07

**Authors:** Simone Cupido, Federica Valeri, Stefano Nicoli, Paolo Bargellini, Domenico Caivano, Francesco Birettoni, Andrea Bortolotti, Mark Rishniw, Francesco Porciello

**Affiliations:** 1 Department of Veterinary Medicine, University of Perugia, Via San Costanzo 4, Perugia, 06126 Italy; 2 Touchlabs 3Ddistrict, Bologna, Italy; 3 AniCura Tyrus Veterinary Clinic, Terni, Italy; 4 Department of Clinical Sciences, Cornell University, Ithaca, NY USA

**Keywords:** Cardiology, Vascular surgery, Virtual reality, Augmented reality, Canine

## Abstract

Extended reality includes both virtual and augmented realities. In virtual reality objects are rendered in an artificial environment where the user can move and interact with a head mounted display. In augmented reality virtual objects are superimposed to real environment enriching it via a head mounted display. In human medicine these technologies have been already used for educational surgical purposes, but remain relatively unknown in veterinary medicine. We report a case of a 1-year-old, female, French bulldog presented for exercise intolerance and dyspnea. Echocardiography showed signs of left ventricular enlargement with reduced fractional shortening and turbulent flow distal to the pulmonary artery bifurcation. Computed tomography revealed a complex vascular network comprising the descending aorta and left pulmonary artery resembling a patent ductus arteriosus. Virtual reality was used for the surgical planning and a left thoracotomy was performed to close the abnormal vessel at the level of the entrance in the left pulmonary artery with augmented reality assistance. No complications were reported during or after the surgery and the dog completely recovered. Echocardiographic findings 3 days, 1 month and 18 months after the surgery demonstrated absence of residual flow and improving ventricular dimensions. To our knowledge this report documents the first use of extended reality for the visualization, planning and execution of the surgical correction of a complex vascular defect in veterinary medicine.

**Supplementary Information:**

The online version contains supplementary material available at 10.1007/s11259-025-10668-1.

## Background

Extended reality is a generic term describing technologies that enhance the reality of our physical world with virtual digital objects and includes virtual reality and augmented reality (Chuah 2019). Virtual reality is a technology that creates a completely artificial virtual environment for the observer to feel in another world (Chuah 2019; Aghapour and Bockstahler 2022). The observer can move and interact with the virtual environment using specific visors and controllers. Augmented reality overlays computer-generated data over the real world to modify, superimpose, or enrich the actual live image (Cao and Cerfolio 2019; Aghapour and Bockstahler 2022). Augmented reality can be generated using specific visors or head mounted display or smartphone and controlled with joystick or hand gestures. Virtual reality can be thought of as a replacement of reality, whereas augmented reality enriches it with additional overlying data. The use of extended reality has been applied to gaming, teaching or biomedical purposes, such as surgery planning and approach (Chuah 2019; Cao and Cerfolio 2019; Shimada et al. 2022; Aghapour and Bockstahler 2022).

In veterinary medicine, most of the published papers describe the potential of extended reality in education and training. Its use in clinical fields has been reported in animal models for the localization and surgical removal of pulmonary nodules (Li et al. 2021; Peng et al. 2021) and during laparoscopic procedures (Araujo et al. 2014; Luo et al. 2020; Adballah et al. 2022). To our knowledge, only one veterinary report exists describing a prototype device for surgical planning and approach; the clinical significance of this study was its ability to project and superimpose the three-dimensional computed tomographic scans of the patient during surgery in the field of view (Shimada et al. 2022).

Therefore, we report the first use of extended reality for the visualization, planning and execution of a surgical correction of a complex vascular defect in a dog.

## Case presentation

**Fig. 1:**
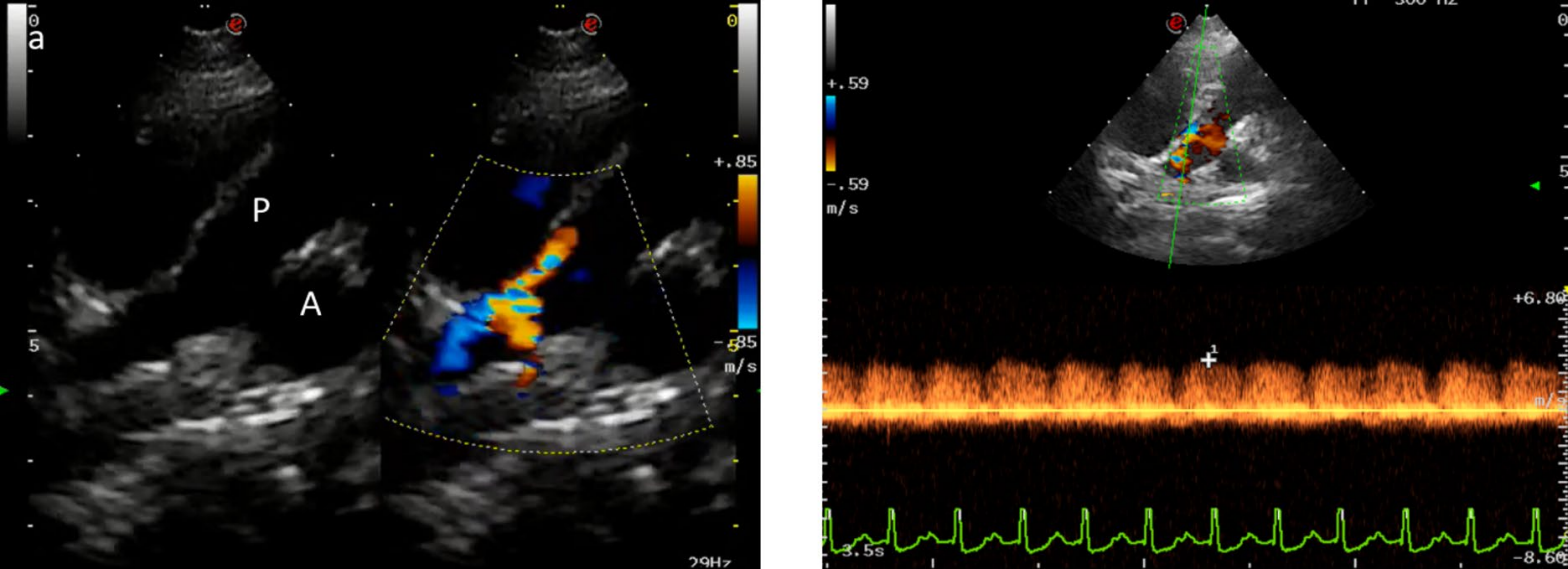
Echocardiographic images of the shunting vessel. **a **Colour doppler aside B-mode image from modified left parasternal view showing an abnormal blood flow in the pulmonary artery. **b** Spectral Doppler through the apparent ostium revealed continuous blood flow. PA, Pulmonary Artery; Ao, Aorta

**Fig. 2:**
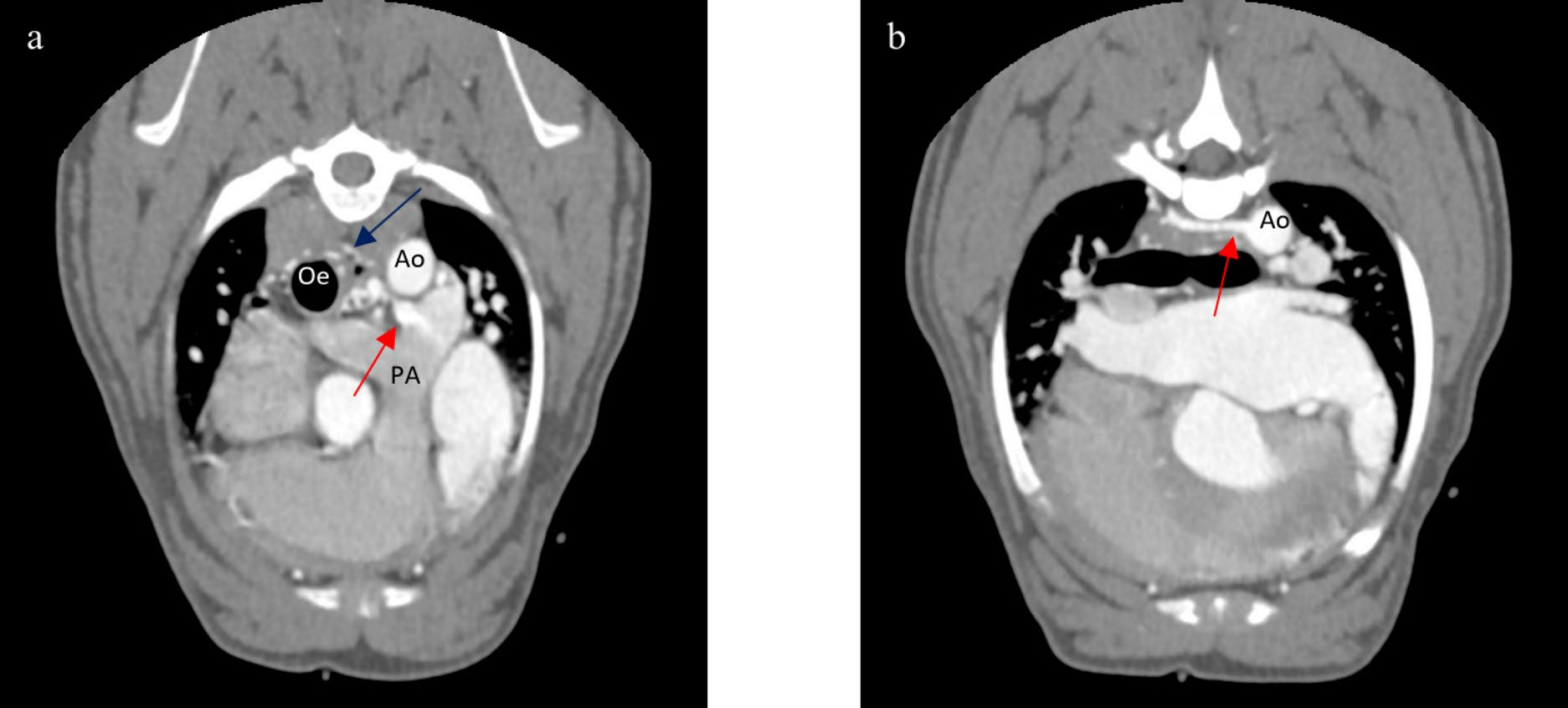
Computed tomographic images contrast enhanced showing the abnormal intrathoracic vessels. **a **Abnormal contrast medium flowing in the left pulmonary artery (red arrow) and vascular network around the oesophagus (blue arrow) are evident. **b **One of three abnormal intercostal arteries arising from the descending aorta (red arrow). PA, Pulmonary Artery; Ao, Aorta; Oe, Oesophagus

A 12-month-old, 11 kg, intact female French Bulldog was referred to the Teaching Veterinary Hospital of Perugia for exercise intolerance and dyspnea. Physical examination revealed a soft machinery murmur best heard on the left second intercostal space area and a moderate left apical systolic murmur. Two-dimensional, mono-dimensional and Doppler echocardiography (MyLab Eight, Esaote, Italy) demonstrated severe left ventricular enlargement (left ventricular internal diameter in diastole normalized, LVIDDN = 1.85) (Cornell et al. 2004; Rishniw and Brown 2022) with decreased fractional shortening (FS = 19%), severely enlarged left atrium (LA/Ao = 2.42) (Rishniw et al. 2019) and a moderate functional mitral regurgitation. Continuous, turbulent blood flow was detected in the main pulmonary artery, arising in a more distal position than typical for a patent ductus arteriosus and flowing perpendicular to the pulmonary artery flow direction (Fig. 1). A “classical” patent ductus arteriosus was not imaged and an ECG-gated computed tomographic angiographic study was recommended. Thoracic ultrasonography showed right sided B-lines consistent with incipient pulmonary oedema. Furosemide (2 mg/kg, BID, PO) and pimobendan (0.25 mg/kg, BID, PO) were administered for the treatment of left congestive heart failure. When the dog was clinically stable, an ECG-gated computed tomographic angiographic study with a 128-row Multi Detector computed tomography unit (Revolution Evo, GE HealthCare, USA; slice thickness = 1,25 mm, WW = 400, WL = 40, mA = 249, kV = 120) was performed. The computed tomography scan demonstrated an anomalous vessel with numerous and tortuous branches arising and connecting with a complex network comprising three right intercostal arteries, the broncho-oesophageal artery and the left pulmonary artery (Fig. 2). This vascular network created a left to right shunt causing left heart volume overload and pre-capillary pulmonary congestion, as in presence of patent ductus arteriosus (Bezuidenhout 1992; Yamane et al. 2001). A diagnosis of a congenital left-to-right systemic to pulmonary artery vascular shunt was made and surgical ligation of the shunting vessels was recommended.

**Fig. 3:**
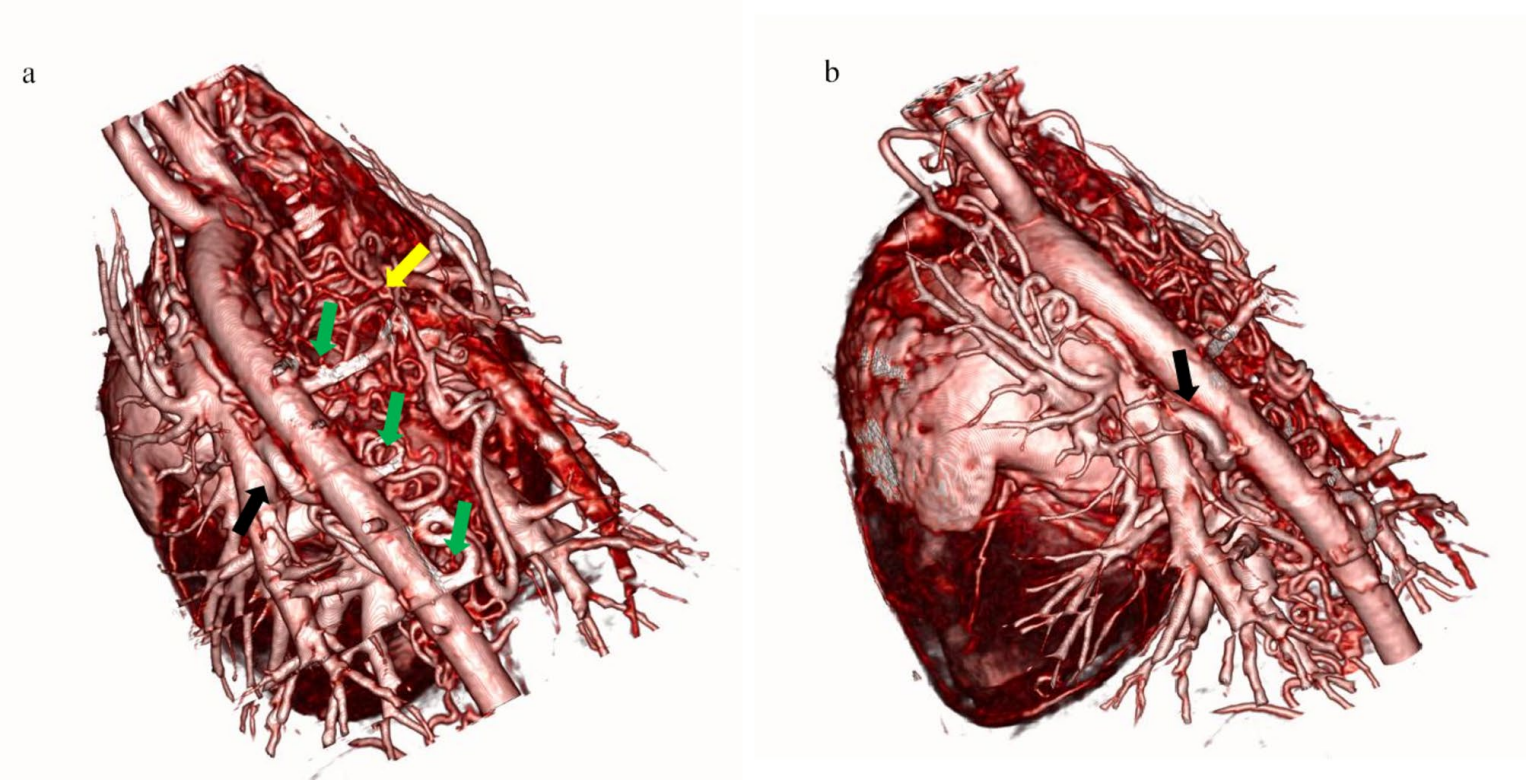
3D-reconstruction model of the dorsal (**a**) and oblique (**b**) aspects of the main intrathoracic vessels arising from the base of the heart. Note the complex vascular network on the right of the descending aorta (yellow arrow), three anomalous intercostal arteries (green arrows), and the abnormal vessel connected to the left pulmonary artery but not to the aorta (black arrow)

Specific software (3D Slicer, Harvard University, USA) permitted the creation of a high-resolution 3D model of the vascular anomaly using computed tomography DICOM data (Fig. 3). The model was displayed in a VR environment using Surgeree software (Brain Storm Dubai, UAE). In this virtual reality environment, surgeons and cardiologists were rendered as avatars by wearing a head-mounted display (Meta Quest 3, Meta, USA) and could manipulate the anatomical model using a controller; it could be possible to shift, resize, rotate and cut the model in a more accurate and intuitive way than through a 2D monitor using a computer. The head-mounted display allowed vocal chat so the specialists could discuss about the surgical planning. It was possible to determine the safest and most effective surgical approach, and the visualization of the exact ligation site needed to close the congenital shunt of the abnormal vessels, where the complex network merged in a single vessel connected to the left pulmonary artery, just above the pulmonary trunk bifurcation. The model studied and elaborated in the virtual reality environment was then displayed in augmented reality through specific glasses (Microsoft Hololens, Washington, USA), that allowed the operator, by hand gestures, to manipulate it.

After formal consent of the owner, patient undergone general anaesthesia using a standard clinical protocol: fasting for 12 h and free access to water; pre-anaesthesia consisted of methadone (0.2 mg/kg) and midazolam (0.3 mg/kg), general anaesthesia was induced with propofol (2 mg/kg) and maintained with isoflurane.

**Fig. 4:**
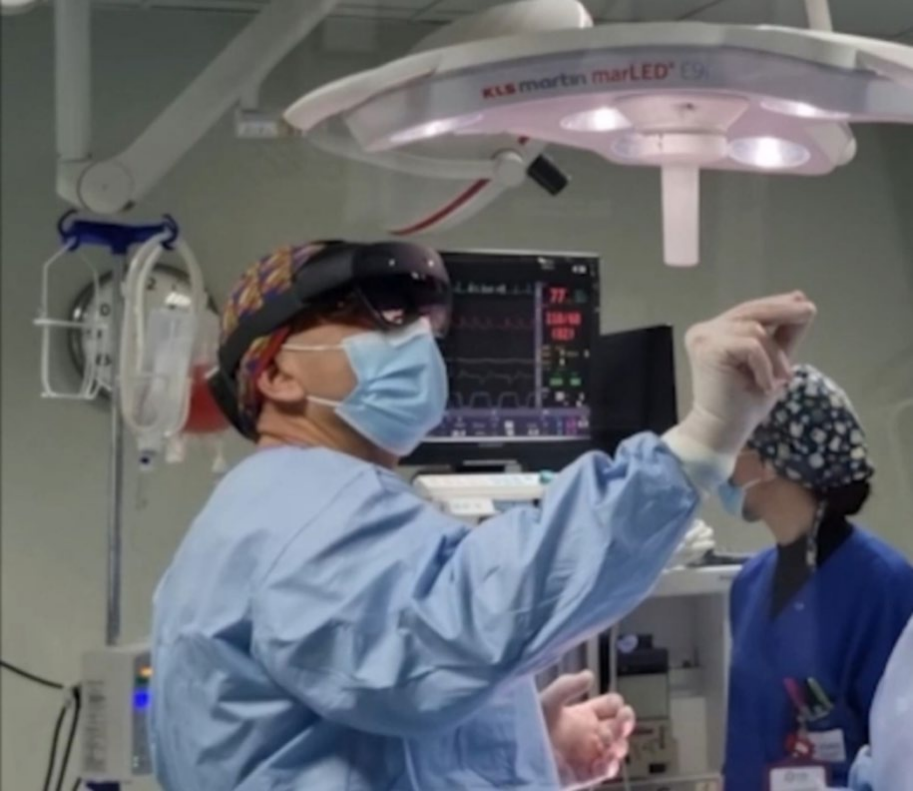
The surgeon wearing the head-mounted display (Hololens) during the procedure while manipulating the augmented reality model by hand gestures

**Fig. 5:**
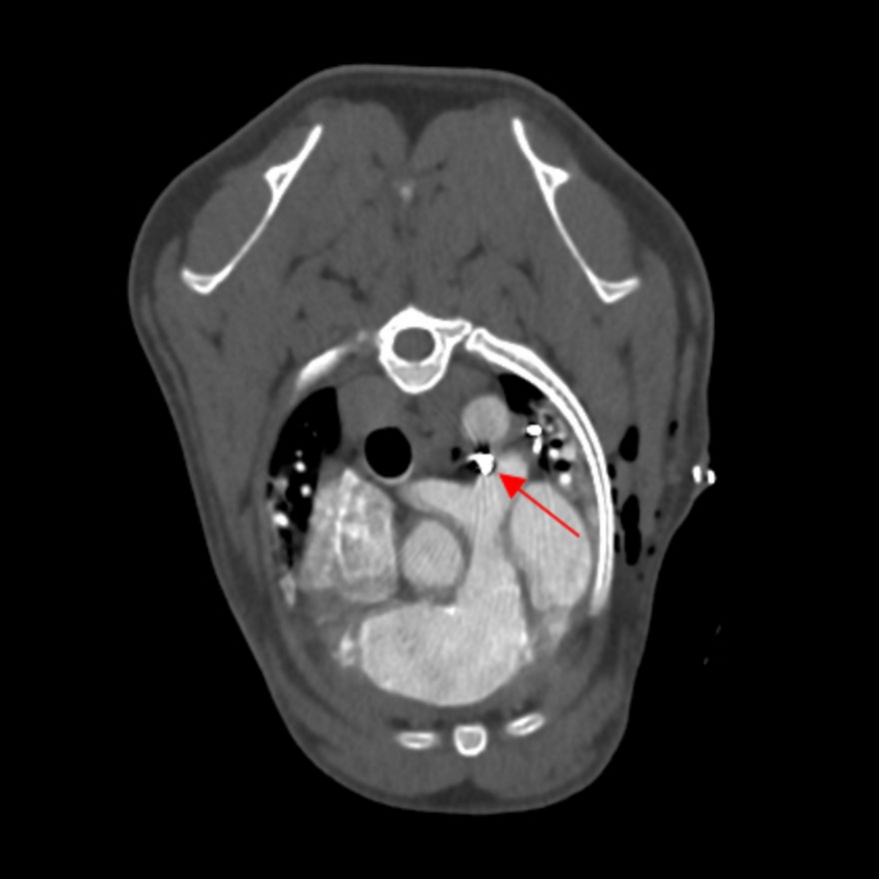
Post-surgical TC scan showing the ligation point at the pulmonary bifurcation (red arrow)

The surgeon (SN) wore the augmented reality head-mounted display (Microsoft Hololens, Washington, USA) which rendered a 3D model of the vasculature and established a virtual connection with cardiologist and radiologist (Fig. 4 and supplementary file 1). It was possible to manipulate, by hand gesture the model aside the real patient during the procedure. With the patient in right lateral recumbency and after the routine aseptically preparation of the operative field, surgical access was performed over the fifth intercostal space to reach the pulmonary trunk. The left pulmonary artery was gently moved laterally using a vessel loop. With the help of the virtual model and the remote real-time collaboration of the different specialists, it was possible to locate the insertion point of the fistula in the pulmonary artery. The fistula was transiently occluded to assess the haemodynamic response. There was an immediate bradycardia, likely as a consequence of a Branham reflex, and after a few minutes the fistula was permanently ligated with prolene suture. The chest wall was closed and sutured and the pneumothorax was resolved by thoracentesis. Another computed tomography scan was performed in general anaesthesia at the end of the surgery, confirming the absence of residual flows (Fig. 5).

No surgical or clinical complications occurred during or after the surgery. The 24-hour follow-up thoracic ultrasound yielded normal findings, so furosemide was discontinued. The three-day follow-up echocardiography showed no residual flow with no significative changes in left ventricular dimensions and slight reduction of left atrium dimension (LA/Ao = 2,1). One month after the surgery the dog showed no clinical signs and the echocardiography demonstrated absence of residual flow with improvement of the left ventricular dimensions (LVIDDN = 1.77, FS = 19.5%) (Cornell et al. 2004; Rishniw and Brown 2022) The eighteen-month follow-up demonstrated further improvement of left ventricular diastolic diameters and fractional shortening (LVVIDN = 1.75, FS = 26.3%) with normal left atrium dimension (LA/Ao = 1.25) (Cornell et al. 2004; Saunders et al. 2014; Rishniw and Brown 2022).

### Discussion and conclusions

In this case report the virtual model allowed a better understanding of the vascular anomaly with the head-mounted display worn before the surgery, visualizing the exact ligation site needed to close the congenital shunting, where the complex network merged in a single vessel connected to the left pulmonary artery, and defining the safest and most effective surgical approach. Furthermore, extended reality and the real-time remote collaboration with the cardiologist and radiologist during the surgery helped localize the exact insertion point of the fistula in the pulmonary artery, which was hidden by a complex network of numerous and tortuous branches (right intercostal arteries, bronchoesophageal artery and left pulmonary artery).

Utility of extended reality has been described in human medicine for surgery purposes over the last decade. It proved itself to be potentially useful for improving operating experience, decreasing operative time, lowering rate of surgical mistakes and eventually decreasing overall costs for healthcare systems (Yoon et al. 2018). Augmented reality has proven very useful in the fields of surgical oncology (Barcali et al. 2022), orthopaedics (Vávra et al. 2017; McKnight et al. 2020; Barcali et al. 2022), spinal surgery(Vávra et al. 2017) and neurosurgery(Vávra et al. 2017; Lungu et al. 2021) in human patients. The ability to visualize virtual images during surgery allows a significant reduction in the duration of the surgical procedure during tumour resection. This is possible due to the accurate visualization of the anatomy and vascularization of the pathological structure, both before and during the procedure, that need to be removed. This helps to properly evaluate the margins of the lesion, reducing the surgical invasiveness and intraoperative complications (Barcali et al. 2022). In orthopaedics, extended reality allows the reconstruction of complex lesions digitally, optimizing planning and the surgical approach (Yoon et al. 2018; Barcali et al. 2022). In neurosurgery, extended reality helps to drastically reduce the margin of error and local invasiveness of the procedure, and simultaneously reduces exposure to ionizing radiation in surgical procedures involving x-rays for both the patient and the surgical team (Vávra et al. 2017; McKnight et al. 2020). In surgical training, extended reality improves learning curves of surgeons, surgical residents and students in surgical techniques such as laparoscopic surgery (McKnight et al. 2020; Lungu et al. 2021; Barcali et al. 2022). This helps to reduce learning times and allowing unlimited practice and correction of errors without compromising patients (Buote et al. 2024a, b). Early applications of extended reality in the medical field showed limitations such as quality image and device dimension; however, recent advances in computer power, image quality and head-mounted display technology have produced devices compact enough for use in a clinical environment with an image quality similar to reality (Vávra et al. 2017; Eckert et al. 2019; Cao and Cerfolio 2019).

In veterinary medicine there are no clinical reports on extended reality application. Most of the published papers describe the potential value of extended reality in the education fields. Some authors described that the use of augmented reality generates similar results, in terms of learning, as traditional methods with books and 2D images, but increases enthusiasm in the study of subjects such as anatomy (Little et al. 2021; Jiang et al. 2024) and pathology (Atmaca and Terzi 2021). Moreover, the use of extended reality can virtually eliminate the use of cadavers for teaching purposes, providing an appropriate alternative to animal studies to comply with the “replacement, reduction, refinement” principle (Eckert et al. 2019; Atmaca and Terzi 2021; Little et al. 2021; Xu et al. 2021; Jiang et al. 2024). Recently, the COVID-19 pandemic has resulted in new demands on the use of new remote learning technologies including extended reality (Xu et al. 2021). The use of extended reality has been tested in a group of veterinary students to investigate whether the preoperative virtual reality simulation can improve the students’ performance in ovariohysterectomy surgery on dogs. However, no significant difference was reported between this group and the control group regarding surgical performance scores and time (Hunt et al. 2020). The application of augmented reality in the veterinary surgery has been also described in canine and porcine models. In a canine model with an artificially created lung tumour, augmented reality allowed successful localization and subsequent excision of the neoplasm with good results and without post-operative complications (Li et al. 2021; Aghapour and Bockstahler 2022). Similar results were obtained in a porcine model in the excision of single pulmonary nodules (Peng et al. 2021). The authors proposed that this method might be used in the future for surgical treatment of early-stage lung cancer (Li et al. 2021; Peng et al. 2021). Unfortunately, clinical trials evaluating the difference in terms of feasibility and time duration in real surgical procedures with and without extended reality are unavailable in the literature. Some authors have also investigated the use of extended reality during laparoscopic procedures in animal models.

Luo et al. (2020) (Luo et al. 2020) used augmented reality to navigate liver resection on ex vivo porcine liver and in vivo porcine: authors confirmed the validity of both ex and in vivo experiments and established that augmented-reality-assisted laparoscopic liver resection can help in practice models. Araujo et al. (2014) (Araujo et al. 2014) reported that participants who practiced laparoscopic colectomy on the virtual reality simulator prior to the actual surgery performed better on the real porcine model. In another laparoscopic study, Adballah et al. (2022) (Adballah et al. 2022) described the use of augmented-reality-assisted laparoscopic liver resection on ex vivo sheep livers with augmented reality software: authors performed laparoscopic resections of pseudo-tumours created in sheep cadaveric livers and compared the results with standard ultrasonography. They reported that augmented-reality-assisted laparoscopy provides precise resection margins, which could reduce surgical errors (Araujo et al. 2014).

Clinical trials are needed to verify the effectiveness and safety of extended reality-guided procedures compared to traditional ones. Head-mounted displays are devices that can weigh up to several hundred grams and which intrinsically generate heat. Furthermore, investigators have reported the development of side effects as dizziness, migraines, nausea and vomiting with their use (Vávra et al. 2017; Hunt et al. 2020). In our case the surgical procedure lasted approximately 1 h, and the visor was not used for the entire procedure, therefore it is necessary to evaluate their comfort of use in complex surgical interventions that require more hours to get complete.

The surgical treatment of the complex vascular anomaly described here was technically simple since it required the ligation of just one vessel with a procedure similar to that of traditional PDA closure. However, in our case a complex network of numerous and tortuous branches hid the exact insertion point of the fistula in the pulmonary artery. During the surgery, the surgeon wore the device to help identify the ligation point that had been previously pinpointed during the pre-surgical briefing with the cardiologist and radiologist. This helped localize the exact insertion point of the fistula in the pulmonary artery and subjectively reduce the surgical time.

The limitations of the use of the extended reality in surgical procedures are the relatively high initial cost of the head mounted display and the time required for the creation of the high-resolution 3D anatomical model.

In conclusion, extended reality is a rapidly developing technology that is gaining attention in veterinary medicine. Our case report demonstrates that this technology can also be used effectively in clinical practice opening new frontiers in the diagnostic and therapeutic aspects of veterinary medicine, where the clinical applications have yet to be widely explored.

## Supplementary Information

Supplementary Material 1.

## Data Availability

No datasets were generated or analysed during the current study.
